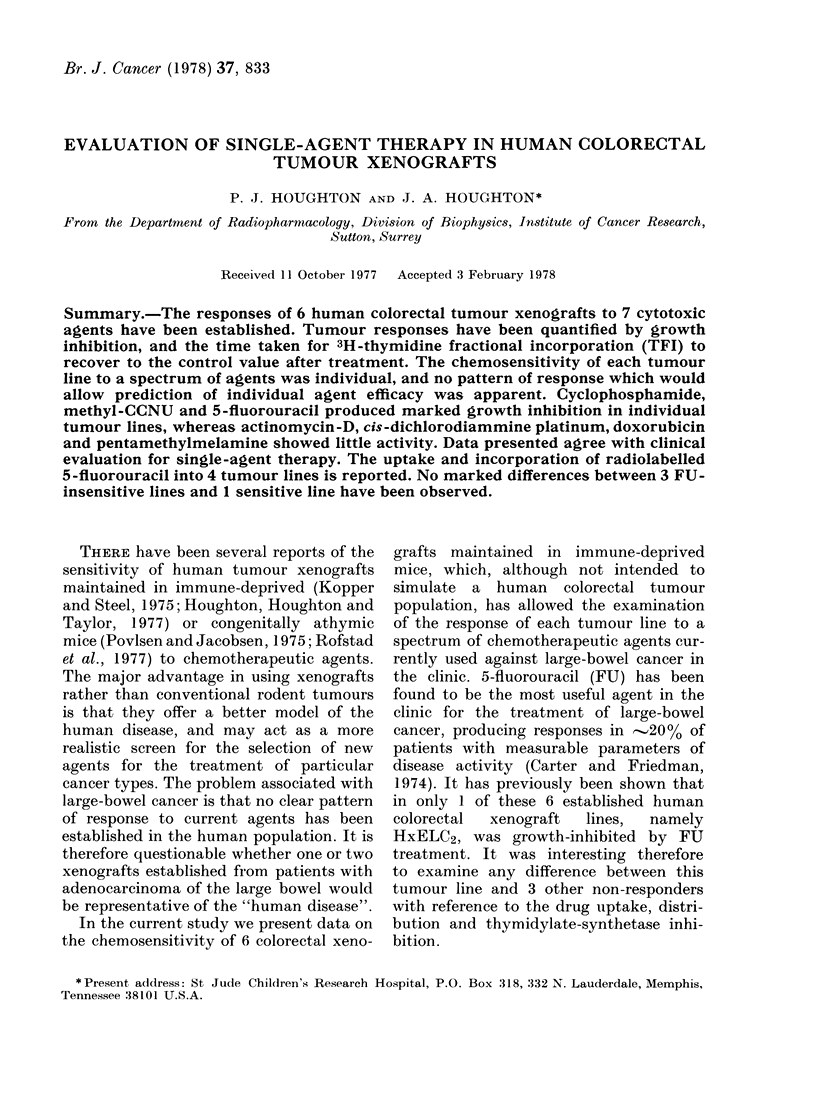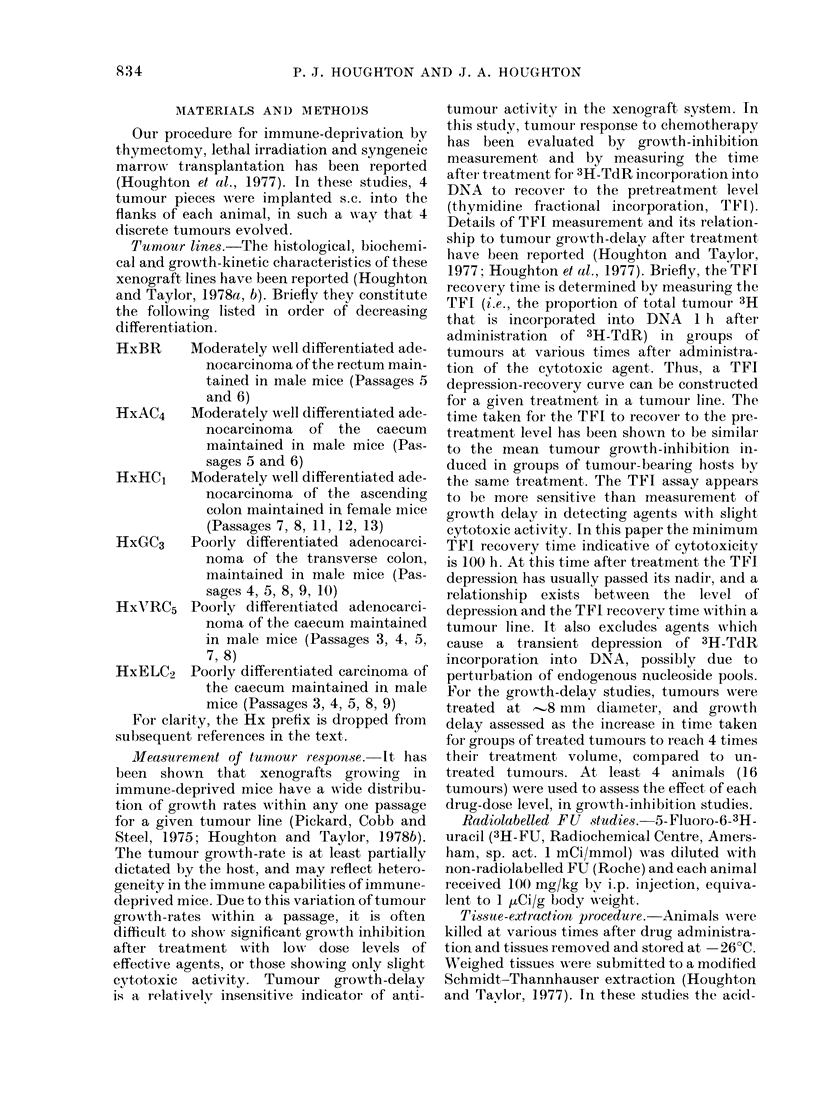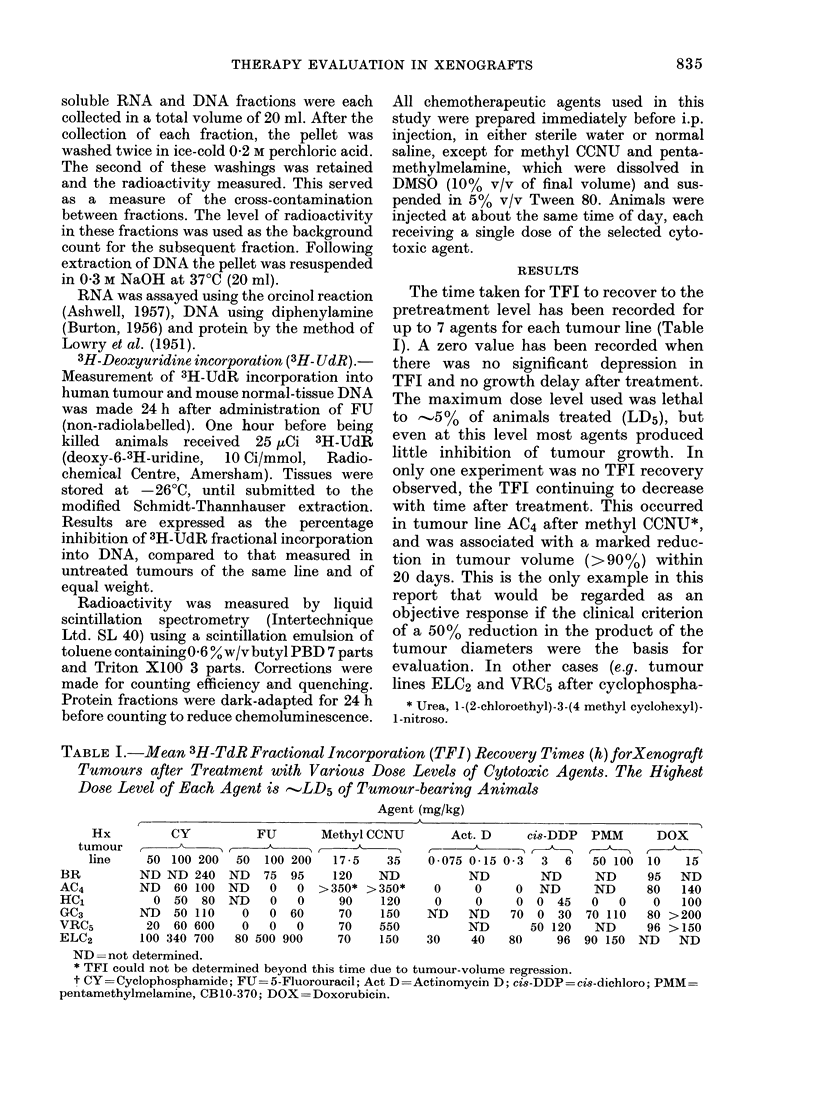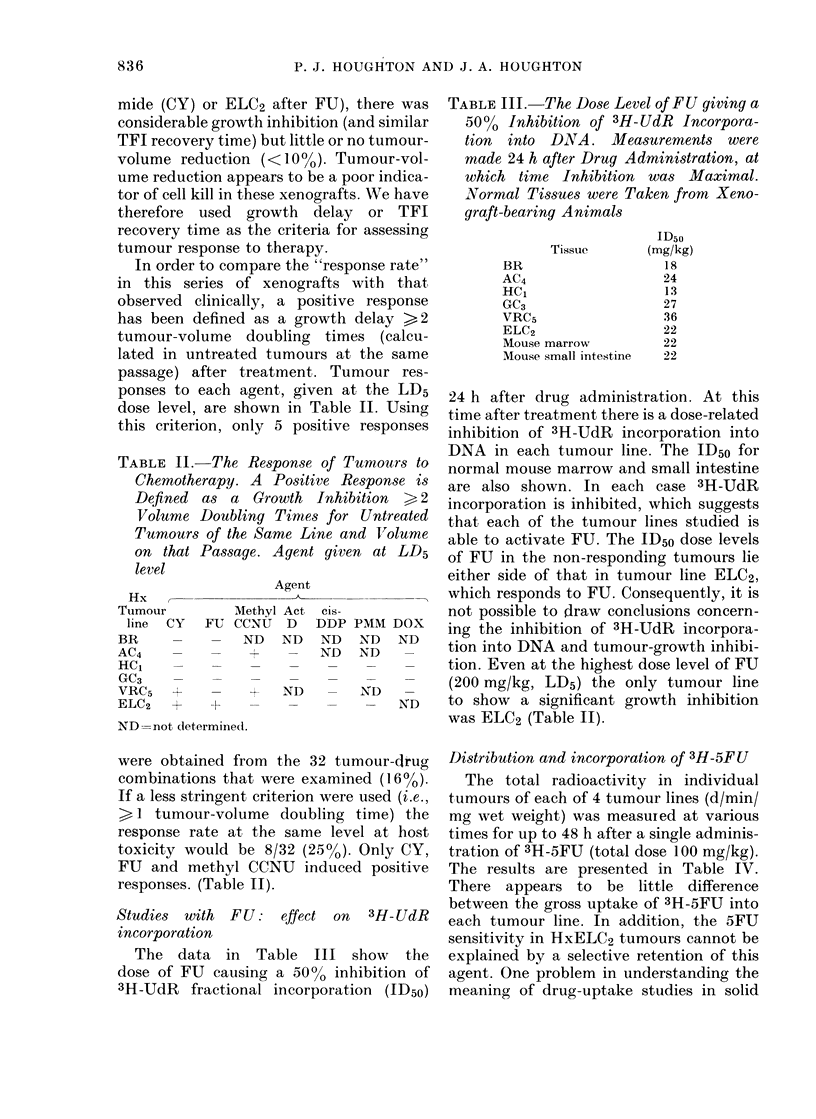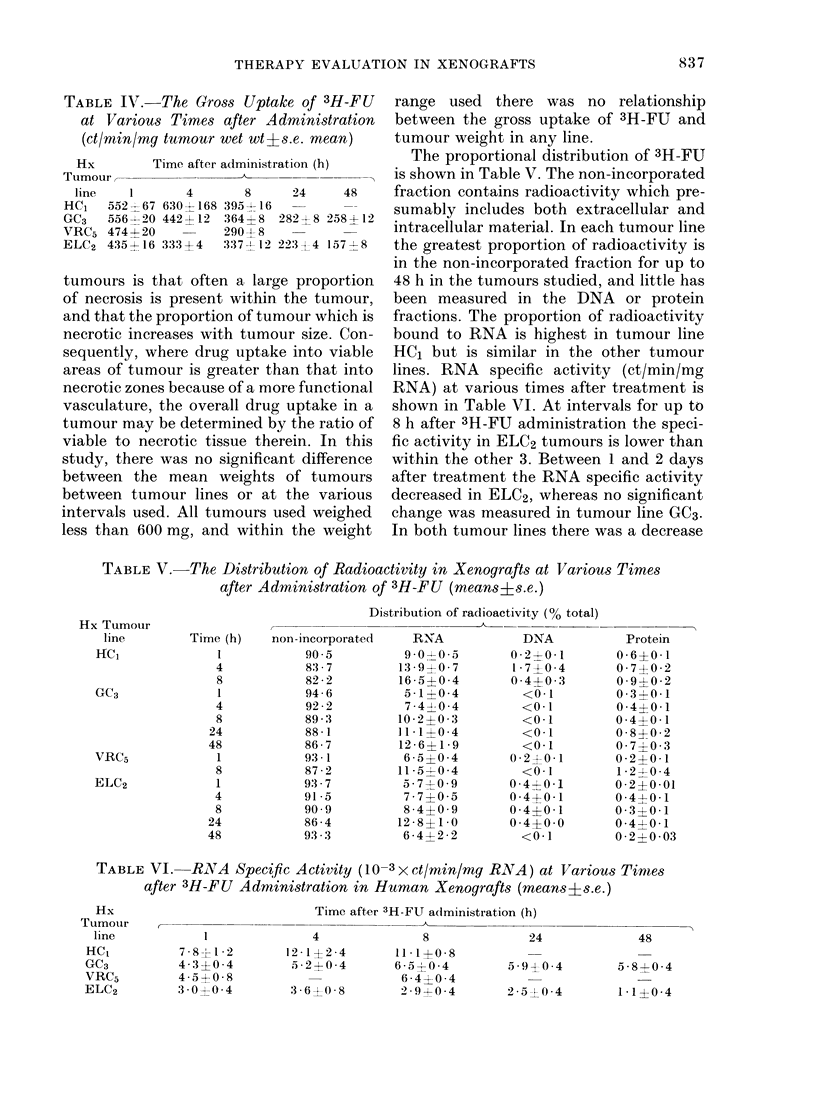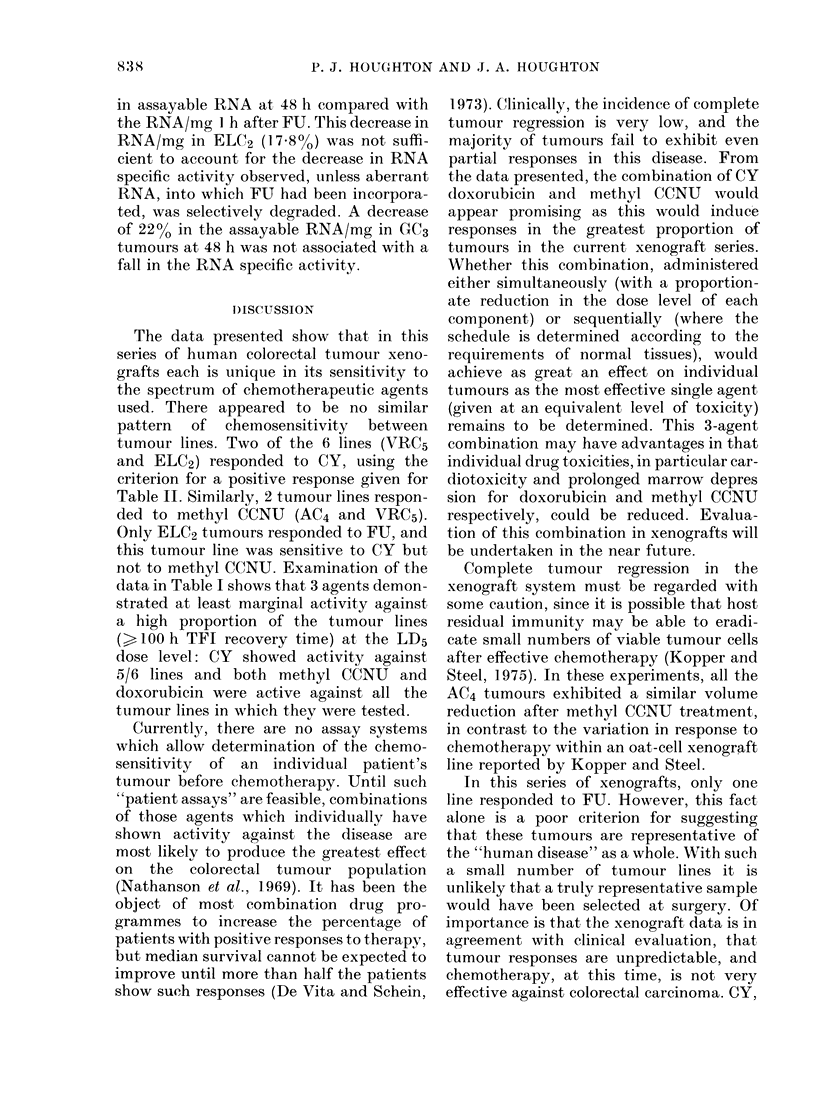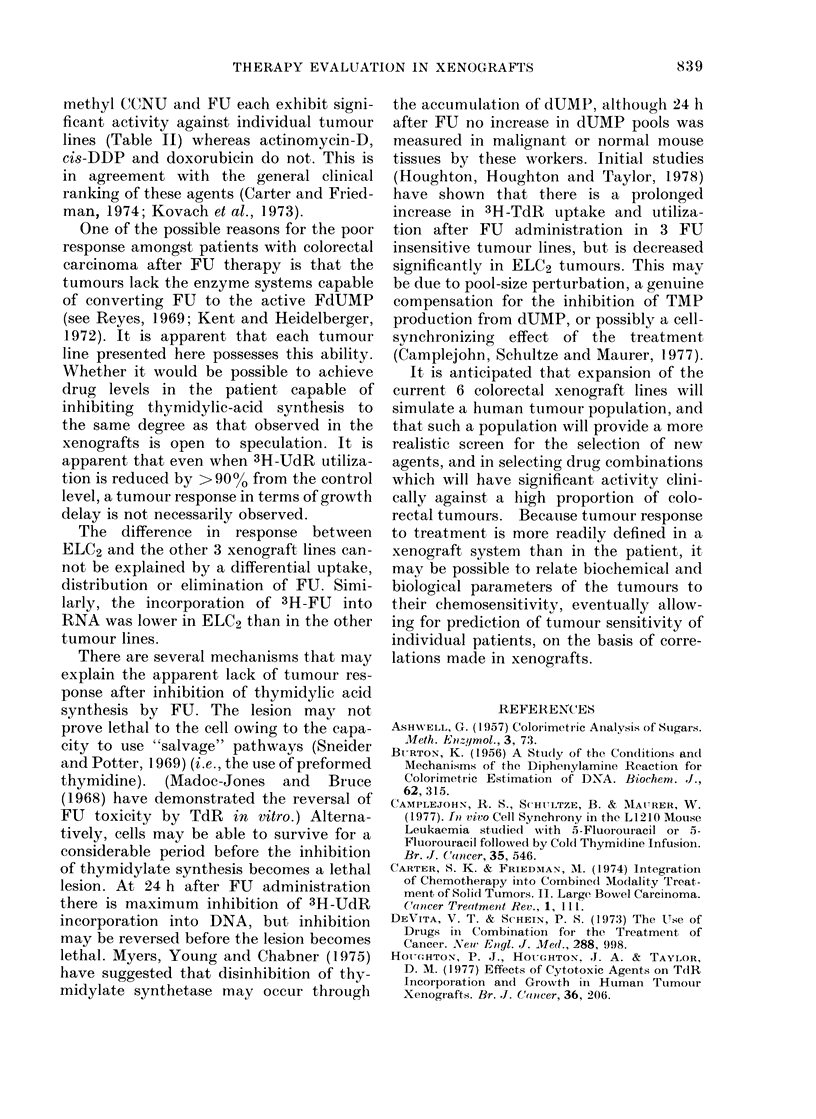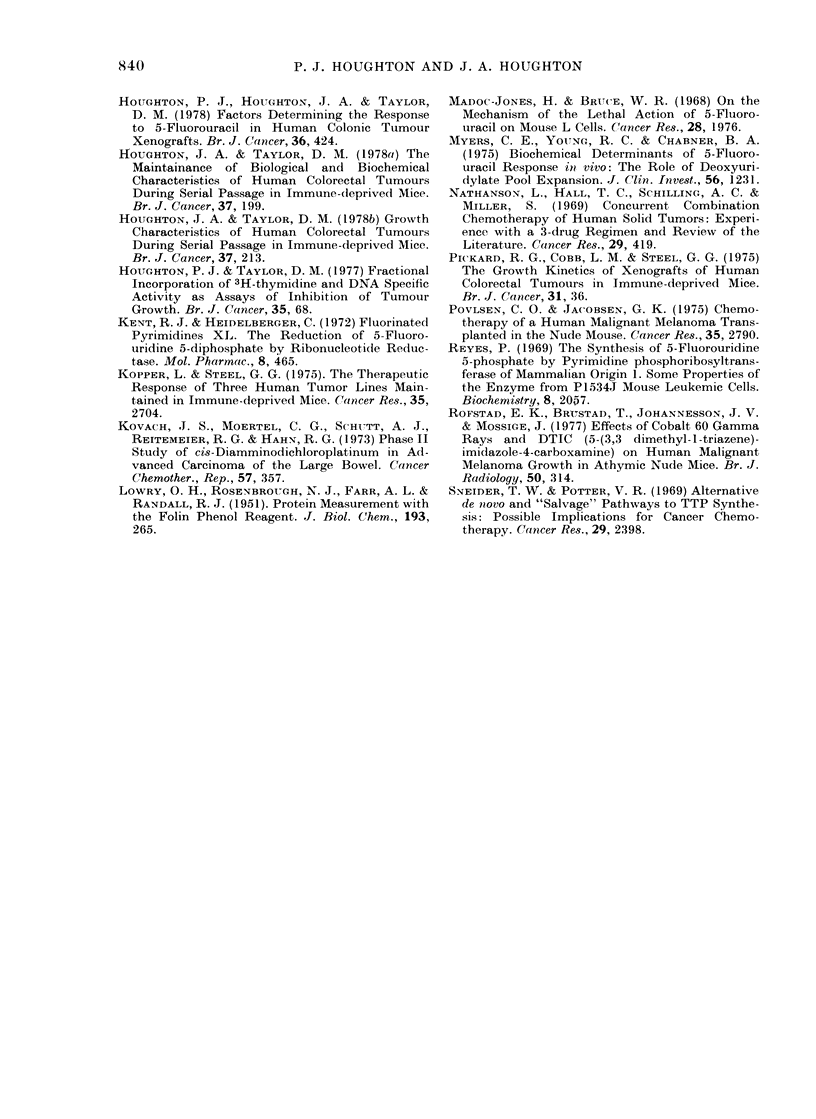# Evaluation of single-agent therapy in human colorectal tumour xenografts.

**DOI:** 10.1038/bjc.1978.122

**Published:** 1978-05

**Authors:** P. J. Houghton, J. A. Houghton

## Abstract

The response of 6 human colorectal tumour xenografts to 7 cytotoxic agents have been established. Tumour responses have been quantified by growth inhibition, and the time taken for 3H-thymidine fractional incorporation (TFI) to recover to the control value after treatment. The chemosensitivity of each tumour line to a spectrum of agents was individual, and no pattern of response which would allow prediction of individual agent efficacy was apparent. Cyclophosphamide, methyl-CCNU and 5-fluorouracil produced marked growth inhibition in individual tumour lines, whereas actinomycin-D, cis-dichlorodiammine platinum, doxorubicin and pentamethylmelamine showed little activity. Data presented agree with clinical evaluation for single-agent therapy. The uptake and incorporation of radiolabelled 5-fluorouracil into 4 tumour lines is reported. No marked differences between 3 FU-insensitive lines and 1 sensitive line have been observed.


*Br. J. Cancer* (1978) **37**, 833

### EVALUATION OF SINGLE-AGENT THERAPY IN HUMAN COLORECTAL TUMOUR XENOGRAFTS

P. J. HOUGHTON AND J. A. HOUGHTON*

*From the Department of Radiopharmacology, Division of Biophysics, Institute of Cancer Research, Sutton, Surrey*

Received 11 October 1977 Accepted 3 February 1978

**Summary.—The responses of 6 human colorectal tumour xenografts to 7 cytotoxic agents have been established. Tumour responses have been quantified by growth inhibition, and the time taken for $^3$H-thymidine fractional incorporation (TFI) to recover to the control value after treatment. The chemosensitivity of each tumour line to a spectrum of agents was individual, and no pattern of response which would allow prediction of individual agent efficacy was apparent. Cyclophosphamide, methyl-CCNU and 5-fluorouracil produced marked growth inhibition in individual tumour lines, whereas actinomycin-D, *cis*-dichlorodiammine platinum, doxorubicin and pentamethylmelamine showed little activity. Data presented agree with clinical evaluation for single-agent therapy. The uptake and incorporation of radiolabelled 5-fluorouracil into 4 tumour lines is reported. No marked differences between 3 FU-insensitive lines and 1 sensitive line have been observed.**

THERE have been several reports of the sensitivity of human tumour xenografts maintained in immune-deprived (Kopper and Steel, 1975; Houghton, Houghton and Taylor, 1977) or congenitally athymic mice (Povlsen and Jacobsen, 1975; Rofstad *et al.*, 1977) to chemotherapeutic agents. The major advantage in using xenografts rather than conventional rodent tumours is that they offer a better model of the human disease, and may act as a more realistic screen for the selection of new agents for the treatment of particular cancer types. The problem associated with large-bowel cancer is that no clear pattern of response to current agents has been established in the human population. It is therefore questionable whether one or two xenografts established from patients with adenocarcinoma of the large bowel would be representative of the "human disease".

In the current study we present data on the chemosensitivity of 6 colorectal xenografts maintained in immune-deprived mice, which, although not intended to simulate a human colorectal tumour population, has allowed the examination of the response of each tumour line to a spectrum of chemotherapeutic agents currently used against large-bowel cancer in the clinic. 5-fluorouracil (FU) has been found to be the most useful agent in the clinic for the treatment of large-bowel cancer, producing responses in $\sim$20% of patients with measurable parameters of disease activity (Carter and Friedman, 1974). It has previously been shown that in only 1 of these 6 established human colorectal xenograft lines, namely HxELC$_2$, was growth-inhibited by FU treatment. It was interesting therefore to examine any difference between this tumour line and 3 other non-responders with reference to the drug uptake, distribution and thymidylate-synthetase inhibition.

*Present address: St Jude Children's Research Hospital, P.O. Box 318, 332 N. Lauderdale, Memphis, Tennessee 38101 U.S.A.

### MATERIALS AND METHODS

Our procedure for immune-deprivation by thymectomy, lethal irradiation and syngeneic marrow transplantation has been reported (Houghton *et al.*, 1977). In these studies, 4 tumour pieces were implanted s.c. into the flanks of each animal, in such a way that 4 discrete tumours evolved.

*Tumour lines.*—The histological, biochemical and growth-kinetic characteristics of these xenograft lines have been reported (Houghton and Taylor, 1978*a*, *b*). Briefly they constitute the following listed in order of decreasing differentiation.

| | |
|---|---|
| $HxBR$ | Moderately well differentiated adenocarcinoma of the rectum maintained in male mice (Passages 5 and 6) |
| $HxAC_4$ | Moderately well differentiated adenocarcinoma of the caecum maintained in male mice (Passages 5 and 6) |
| $HxHC_1$ | Moderately well differentiated adenocarcinoma of the ascending colon maintained in female mice (Passages 7, 8, 11, 12, 13) |
| $HxGC_3$ | Poorly differentiated adenocarcinoma of the transverse colon, maintained in male mice (Passages 4, 5, 8, 9, 10) |
| $HxVRC_5$ | Poorly differentiated adenocarcinoma of the caecum maintained in male mice (Passages 3, 4, 5, 7, 8) |
| $HxELC_2$ | Poorly differentiated carcinoma of the caecum maintained in male mice (Passages 3, 4, 5, 8, 9) |

For clarity, the Hx prefix is dropped from subsequent references in the text.

*Measurement of tumour response.*—It has been shown that xenografts growing in immune-deprived mice have a wide distribution of growth rates within any one passage for a given tumour line (Pickard, Cobb and Steel, 1975; Houghton and Taylor, 1978*b*). The tumour growth-rate is at least partially dictated by the host, and may reflect heterogeneity in the immune capabilities of immune-deprived mice. Due to this variation of tumour growth-rates within a passage, it is often difficult to show significant growth inhibition after treatment with low dose levels of effective agents, or those showing only slight cytotoxic activity. Tumour growth-delay is a relatively insensitive indicator of antitumour activity in the xenograft system. In this study, tumour response to chemotherapy has been evaluated by growth-inhibition measurement and by measuring the time after treatment for $^3$H-TdR incorporation into DNA to recover to the pretreatment level (thymidine fractional incorporation, TFI). Details of TFI measurement and its relationship to tumour growth-delay after treatment have been reported (Houghton and Taylor, 1977; Houghton *et al.*, 1977). Briefly, the TFI recovery time is determined by measuring the TFI (*i.e.*, the proportion of total tumour $^3$H that is incorporated into DNA 1 h after administration of $^3$H-TdR) in groups of tumours at various times after administration of the cytotoxic agent. Thus, a TFI depression-recovery curve can be constructed for a given treatment in a tumour line. The time taken for the TFI to recover to the pretreatment level has been shown to be similar to the mean tumour growth-inhibition induced in groups of tumour-bearing hosts by the same treatment. The TFI assay appears to be more sensitive than measurement of growth delay in detecting agents with slight cytotoxic activity. In this paper the minimum TFI recovery time indicative of cytotoxicity is 100 h. At this time after treatment the TFI depression has usually passed its nadir, and a relationship exists between the level of depression and the TFI recovery time within a tumour line. It also excludes agents which cause a transient depression of $^3$H-TdR incorporation into DNA, possibly due to perturbation of endogenous nucleoside pools. For the growth-delay studies, tumours were treated at $\sim$8 mm diameter, and growth delay assessed as the increase in time taken for groups of treated tumours to reach 4 times their treatment volume, compared to untreated tumours. At least 4 animals (16 tumours) were used to assess the effect of each drug-dose level, in growth-inhibition studies.

*Radiolabelled FU studies.*—5-Fluoro-6-$^3$H-uracil ($^3$H-FU, Radiochemical Centre, Amersham, sp. act. 1 mCi/mmol) was diluted with non-radiolabelled FU (Roche) and each animal received 100 mg/kg by i.p. injection, equivalent to 1 $\mu$Ci/g body weight.

*Tissue-extraction procedure.*—Animals were killed at various times after drug administration and tissues removed and stored at $-26$°C. Weighed tissues were submitted to a modified Schmidt–Thannhauser extraction (Houghton and Taylor, 1977). In these studies the acidsoluble RNA and DNA fractions were each collected in a total volume of 20 ml. After the collection of each fraction, the pellet was washed twice in ice-cold 0·2 M perchloric acid. The second of these washings was retained and the radioactivity measured. This served as a measure of the cross-contamination between fractions. The level of radioactivity in these fractions was used as the background count for the subsequent fraction. Following extraction of DNA the pellet was resuspended in 0·3 M NaOH at 37°C (20 ml).

RNA was assayed using the orcinol reaction (Ashwell, 1957), DNA using diphenylamine (Burton, 1956) and protein by the method of Lowry *et al.* (1951).

*$^3$H-Deoxyuridine incorporation* ($^3$*H-UdR*).—Measurement of $^3$H-UdR incorporation into human tumour and mouse normal-tissue DNA was made 24 h after administration of FU (non-radiolabelled). One hour before being killed animals received 25 $\mu$Ci $^3$H-UdR (deoxy-6-$^3$H-uridine, 10 Ci/mmol, Radiochemical Centre, Amersham). Tissues were stored at −26°C, until submitted to the modified Schmidt-Thannhauser extraction. Results are expressed as the percentage inhibition of $^3$H-UdR fractional incorporation into DNA, compared to that measured in untreated tumours of the same line and of equal weight.

Radioactivity was measured by liquid scintillation spectrometry (Intertechnique Ltd. SL 40) using a scintillation emulsion of toluene containing 0·6% w/v butyl PBD 7 parts and Triton X100 3 parts. Corrections were made for counting efficiency and quenching. Protein fractions were dark-adapted for 24 h before counting to reduce chemoluminescence.

All chemotherapeutic agents used in this study were prepared immediately before i.p. injection, in either sterile water or normal saline, except for methyl CCNU and pentamethylmelamine, which were dissolved in DMSO (10% v/v of final volume) and suspended in 5% v/v Tween 80. Animals were injected at about the same time of day, each receiving a single dose of the selected cytotoxic agent.

### RESULTS

The time taken for TFI to recover to the pretreatment level has been recorded for up to 7 agents for each tumour line (Table I). A zero value has been recorded when there was no significant depression in TFI and no growth delay after treatment. The maximum dose level used was lethal to ~5% of animals treated ($LD_5$), but even at this level most agents produced little inhibition of tumour growth. In only one experiment was no TFI recovery observed, the TFI continuing to decrease with time after treatment. This occurred in tumour line $AC_4$ after methyl CCNU*, and was associated with a marked reduction in tumour volume (>90%) within 20 days. This is the only example in this report that would be regarded as an objective response if the clinical criterion of a 50% reduction in the product of the tumour diameters were the basis for evaluation. In other cases (*e.g.* tumour lines $ELC_2$ and $VRC_5$ after cyclophospha-

* Urea, 1-(2-chloroethyl)-3-(4 methyl cyclohexyl)-1-nitroso.

TABLE I.—*Mean $^3$H-TdR Fractional Incorporation (TFI) Recovery Times (h) for Xenograft Tumours after Treatment with Various Dose Levels of Cytotoxic Agents. The Highest Dose Level of Each Agent is ~$LD_5$ of Tumour-bearing Animals*

| Hx tumour line | Agent (mg/kg) | | | | | | | | | | | | | | | | |
|---|---|---|---|---|---|---|---|---|---|---|---|---|---|---|---|---|---|
| | CY | | | FU | | | Methyl CCNU | | Act. D | | | *cis*-DDP | | PMM | | DOX | |
| | 50 | 100 | 200 | 50 | 100 | 200 | 17·5 | 35 | 0·075 | 0·15 | 0·3 | 3 | 6 | 50 | 100 | 10 | 15 |
| BR | ND | ND | 240 | ND | 75 | 95 | 120 | ND | | ND | | ND | | ND | | 95 | ND |
| $AC_4$ | ND | 60 | 100 | ND | 0 | 0 | >350* | >350* | 0 | 0 | 0 | ND | | ND | | 80 | 140 |
| $HC_1$ | 0 | 50 | 80 | ND | 0 | 0 | 90 | 120 | 0 | 0 | 0 | 0 | 45 | 0 | 0 | 0 | 100 |
| $GC_3$ | ND | 50 | 110 | 0 | 0 | 60 | 70 | 150 | ND | ND | 70 | 0 | 30 | 70 | 110 | 80 | >200 |
| $VRC_5$ | 20 | 60 | 600 | 0 | 0 | 0 | 70 | 550 | | ND | | 50 | 120 | ND | | 96 | >150 |
| $ELC_2$ | 100 | 340 | 700 | 80 | 500 | 900 | 70 | 150 | 30 | 40 | 80 | | 96 | 90 | 150 | ND | ND |

ND = not determined.

* TFI could not be determined beyond this time due to tumour-volume regression.

† CY = Cyclophosphamide; FU = 5-Fluorouracil; Act D = Actinomycin D; *cis*-DDP = *cis*-dichloro; PMM = pentamethylmelamine, CB10-370; DOX = Doxorubicin.

mide (CY) or $ELC_2$ after FU), there was considerable growth inhibition (and similar TFI recovery time) but little or no tumour-volume reduction (<10%). Tumour-volume reduction appears to be a poor indicator of cell kill in these xenografts. We have therefore used growth delay or TFI recovery time as the criteria for assessing tumour response to therapy.

In order to compare the "response rate" in this series of xenografts with that observed clinically, a positive response has been defined as a growth delay $\geqslant 2$ tumour-volume doubling times (calculated in untreated tumours at the same passage) after treatment. Tumour responses to each agent, given at the $LD_5$ dose level, are shown in Table II. Using this criterion, only 5 positive responses were obtained from the 32 tumour-drug combinations that were examined (16%). If a less stringent criterion were used (*i.e.*, $\geqslant 1$ tumour-volume doubling time) the response rate at the same level at host toxicity would be 8/32 (25%). Only CY, FU and methyl CCNU induced positive responses. (Table II).

TABLE II.—*The Response of Tumours to Chemotherapy. A Positive Response is Defined as a Growth Inhibition $\geqslant 2$ Volume Doubling Times for Untreated Tumours of the Same Line and Volume on that Passage. Agent given at $LD_5$ level*

| Hx Tumour line | Agent CY | FU | Methyl CCNU | Act D | cis-DDP | PMM | DOX |
|---|---|---|---|---|---|---|---|
| BR | – | – | ND | ND | ND | ND | ND |
| $AC_4$ | – | – | + | – | ND | ND | – |
| $HC_1$ | – | – | – | – | – | – | – |
| $GC_3$ | – | – | – | – | – | – | – |
| $VRC_5$ | + | – | + | ND | – | ND | – |
| $ELC_2$ | + | + | – | – | – | – | ND |

ND = not determined.

*Studies with FU: effect on $^3H$-UdR incorporation*

The data in Table III show the dose of FU causing a 50% inhibition of $^3$H-UdR fractional incorporation ($ID_{50}$) 24 h after drug administration. At this time after treatment there is a dose-related inhibition of $^3$H-UdR incorporation into DNA in each tumour line. The $ID_{50}$ for normal mouse marrow and small intestine are also shown. In each case $^3$H-UdR incorporation is inhibited, which suggests that each of the tumour lines studied is able to activate FU. The $ID_{50}$ dose levels of FU in the non-responding tumours lie either side of that in tumour line $ELC_2$, which responds to FU. Consequently, it is not possible to draw conclusions concerning the inhibition of $^3$H-UdR incorporation into DNA and tumour-growth inhibition. Even at the highest dose level of FU (200 mg/kg, $LD_5$) the only tumour line to show a significant growth inhibition was $ELC_2$ (Table II).

TABLE III.—*The Dose Level of FU giving a 50% Inhibition of $^3H$-UdR Incorporation into DNA. Measurements were made 24 h after Drug Administration, at which time Inhibition was Maximal. Normal Tissues were Taken from Xenograft-bearing Animals*

| Tissue | $ID_{50}$ (mg/kg) |
|---|---|
| BR | 18 |
| $AC_4$ | 24 |
| $HC_1$ | 13 |
| $GC_3$ | 27 |
| $VRC_5$ | 36 |
| $ELC_2$ | 22 |
| Mouse marrow | 22 |
| Mouse small intestine | 22 |

*Distribution and incorporation of $^3H$-5FU*

The total radioactivity in individual tumours of each of 4 tumour lines (d/min/mg wet weight) was measured at various times for up to 48 h after a single administration of $^3$H-5FU (total dose 100 mg/kg). The results are presented in Table IV. There appears to be little difference between the gross uptake of $^3$H-5FU into each tumour line. In addition, the 5FU sensitivity in $HxELC_2$ tumours cannot be explained by a selective retention of this agent. One problem in understanding the meaning of drug-uptake studies in solid

TABLE IV.—*The Gross Uptake of $^3$H-FU at Various Times after Administration (ct/min/mg tumour wet wt±s.e. mean)*

| Hx Tumour line | Time after administration (h) 1 | 4 | 8 | 24 | 48 |
|---|---|---|---|---|---|
| $HC_1$ | 552±67 | 630±168 | 395±16 | — | — |
| $GC_3$ | 556±20 | 442±12 | 364±8 | 282±8 | 258±12 |
| $VRC_5$ | 474±20 | — | 290±8 | — | — |
| $ELC_2$ | 435±16 | 333±4 | 337±12 | 223±4 | 157±8 |

tumours is that often a large proportion of necrosis is present within the tumour, and that the proportion of tumour which is necrotic increases with tumour size. Consequently, where drug uptake into viable areas of tumour is greater than that into necrotic zones because of a more functional vasculature, the overall drug uptake in a tumour may be determined by the ratio of viable to necrotic tissue therein. In this study, there was no significant difference between the mean weights of tumours between tumour lines or at the various intervals used. All tumours used weighed less than 600 mg, and within the weight range used there was no relationship between the gross uptake of $^3$H-FU and tumour weight in any line.

The proportional distribution of $^3$H-FU is shown in Table V. The non-incorporated fraction contains radioactivity which presumably includes both extracellular and intracellular material. In each tumour line the greatest proportion of radioactivity is in the non-incorporated fraction for up to 48 h in the tumours studied, and little has been measured in the DNA or protein fractions. The proportion of radioactivity bound to RNA is highest in tumour line $HC_1$ but is similar in the other tumour lines. RNA specific activity (ct/min/mg RNA) at various times after treatment is shown in Table VI. At intervals for up to 8 h after $^3$H-FU administration the specific activity in $ELC_2$ tumours is lower than within the other 3. Between 1 and 2 days after treatment the RNA specific activity decreased in $ELC_2$, whereas no significant change was measured in tumour line $GC_3$. In both tumour lines there was a decrease

TABLE V.—*The Distribution of Radioactivity in Xenografts at Various Times after Administration of $^3$H-FU (means±s.e.)*

| Hx Tumour line | Time (h) | Distribution of radioactivity (% total) non-incorporated | RNA | DNA | Protein |
|---|---|---|---|---|---|
| $HC_1$ | 1 | 90·5 | 9·0±0·5 | 0·2±0·1 | 0·6±0·1 |
| | 4 | 83·7 | 13·9±0·7 | 1·7±0·4 | 0·7±0·2 |
| | 8 | 82·2 | 16·5±0·4 | 0·4±0·3 | 0·9±0·2 |
| $GC_3$ | 1 | 94·6 | 5·1±0·4 | <0·1 | 0·3±0·1 |
| | 4 | 92·2 | 7·4±0·4 | <0·1 | 0·4±0·1 |
| | 8 | 89·3 | 10·2±0·3 | <0·1 | 0·4±0·1 |
| | 24 | 88·1 | 11·1±0·4 | <0·1 | 0·8±0·2 |
| | 48 | 86·7 | 12·6±1·9 | <0·1 | 0·7±0·3 |
| $VRC_5$ | 1 | 93·1 | 6·5±0·4 | 0·2±0·1 | 0·2±0·1 |
| | 8 | 87·2 | 11·5±0·4 | <0·1 | 1·2±0·4 |
| $ELC_2$ | 1 | 93·7 | 5·7±0·9 | 0·4±0·1 | 0·2±0·01 |
| | 4 | 91·5 | 7·7±0·5 | 0·4±0·1 | 0·4±0·1 |
| | 8 | 90·9 | 8·4±0·9 | 0·4±0·1 | 0·3±0·1 |
| | 24 | 86·4 | 12·8±1·0 | 0·4±0·0 | 0·4±0·1 |
| | 48 | 93·3 | 6·4±2·2 | <0·1 | 0·2±0·03 |

TABLE VI.—*RNA Specific Activity ($10^{-3}\times$ct/min/mg RNA) at Various Times after $^3$H-FU Administration in Human Xenografts (means±s.e.)*

| Hx Tumour line | Time after $^3$H-FU administration (h) 1 | 4 | 8 | 24 | 48 |
|---|---|---|---|---|---|
| $HC_1$ | 7·8±1·2 | 12·1±2·4 | 11·1±0·8 | — | — |
| $GC_3$ | 4·3±0·4 | 5·2±0·4 | 6·5±0·4 | 5·9±0·4 | 5·8±0·4 |
| $VRC_5$ | 4·5±0·8 | — | 6·4±0·4 | — | — |
| $ELC_2$ | 3·0±0·4 | 3·6±0·8 | 2·9±0·4 | 2·5±0·4 | 1·1±0·4 |

in assayable RNA at 48 h compared with the RNA/mg 1 h after FU. This decrease in RNA/mg in $ELC_2$ (17·8%) was not sufficient to account for the decrease in RNA specific activity observed, unless aberrant RNA, into which FU had been incorporated, was selectively degraded. A decrease of 22% in the assayable RNA/mg in $GC_3$ tumours at 48 h was not associated with a fall in the RNA specific activity.

### DISCUSSION

The data presented show that in this series of human colorectal tumour xenografts each is unique in its sensitivity to the spectrum of chemotherapeutic agents used. There appeared to be no similar pattern of chemosensitivity between tumour lines. Two of the 6 lines ($VRC_5$ and $ELC_2$) responded to CY, using the criterion for a positive response given for Table II. Similarly, 2 tumour lines responded to methyl CCNU ($AC_4$ and $VRC_5$). Only $ELC_2$ tumours responded to FU, and this tumour line was sensitive to CY but not to methyl CCNU. Examination of the data in Table I shows that 3 agents demonstrated at least marginal activity against a high proportion of the tumour lines ($\geqslant$100 h TFI recovery time) at the $LD_5$ dose level: CY showed activity against 5/6 lines and both methyl CCNU and doxorubicin were active against all the tumour lines in which they were tested.

Currently, there are no assay systems which allow determination of the chemosensitivity of an individual patient's tumour before chemotherapy. Until such "patient assays" are feasible, combinations of those agents which individually have shown activity against the disease are most likely to produce the greatest effect on the colorectal tumour population (Nathanson *et al.*, 1969). It has been the object of most combination drug programmes to increase the percentage of patients with positive responses to therapy, but median survival cannot be expected to improve until more than half the patients show such responses (De Vita and Schein, 1973). Clinically, the incidence of complete tumour regression is very low, and the majority of tumours fail to exhibit even partial responses in this disease. From the data presented, the combination of CY doxorubicin and methyl CCNU would appear promising as this would induce responses in the greatest proportion of tumours in the current xenograft series. Whether this combination, administered either simultaneously (with a proportionate reduction in the dose level of each component) or sequentially (where the schedule is determined according to the requirements of normal tissues), would achieve as great an effect on individual tumours as the most effective single agent (given at an equivalent level of toxicity) remains to be determined. This 3-agent combination may have advantages in that individual drug toxicities, in particular cardiotoxicity and prolonged marrow depres sion for doxorubicin and methyl CCNU respectively, could be reduced. Evaluation of this combination in xenografts will be undertaken in the near future.

Complete tumour regression in the xenograft system must be regarded with some caution, since it is possible that host residual immunity may be able to eradicate small numbers of viable tumour cells after effective chemotherapy (Kopper and Steel, 1975). In these experiments, all the $AC_4$ tumours exhibited a similar volume reduction after methyl CCNU treatment, in contrast to the variation in response to chemotherapy within an oat-cell xenograft line reported by Kopper and Steel.

In this series of xenografts, only one line responded to FU. However, this fact alone is a poor criterion for suggesting that these tumours are representative of the "human disease" as a whole. With such a small number of tumour lines it is unlikely that a truly representative sample would have been selected at surgery. Of importance is that the xenograft data is in agreement with clinical evaluation, that tumour responses are unpredictable, and chemotherapy, at this time, is not very effective against colorectal carcinoma. CY, methyl CCNU and FU each exhibit significant activity against individual tumour lines (Table II) whereas actinomycin-D, *cis*-DDP and doxorubicin do not. This is in agreement with the general clinical ranking of these agents (Carter and Friedman, 1974; Kovach *et al.*, 1973).

One of the possible reasons for the poor response amongst patients with colorectal carcinoma after FU therapy is that the tumours lack the enzyme systems capable of converting FU to the active FdUMP (see Reyes, 1969; Kent and Heidelberger, 1972). It is apparent that each tumour line presented here possesses this ability. Whether it would be possible to achieve drug levels in the patient capable of inhibiting thymidylic-acid synthesis to the same degree as that observed in the xenografts is open to speculation. It is apparent that even when $^3$H-UdR utilization is reduced by $>90\%$ from the control level, a tumour response in terms of growth delay is not necessarily observed.

The difference in response between $ELC_2$ and the other 3 xenograft lines cannot be explained by a differential uptake, distribution or elimination of FU. Similarly, the incorporation of $^3$H-FU into RNA was lower in $ELC_2$ than in the other tumour lines.

There are several mechanisms that may explain the apparent lack of tumour response after inhibition of thymidylic acid synthesis by FU. The lesion may not prove lethal to the cell owing to the capacity to use "salvage" pathways (Sneider and Potter, 1969) (*i.e.*, the use of preformed thymidine). (Madoc-Jones and Bruce (1968) have demonstrated the reversal of FU toxicity by TdR *in vitro*.) Alternatively, cells may be able to survive for a considerable period before the inhibition of thymidylate synthesis becomes a lethal lesion. At 24 h after FU administration there is maximum inhibition of $^3$H-UdR incorporation into DNA, but inhibition may be reversed before the lesion becomes lethal. Myers, Young and Chabner (1975) have suggested that disinhibition of thymidylate synthetase may occur through the accumulation of dUMP, although 24 h after FU no increase in dUMP pools was measured in malignant or normal mouse tissues by these workers. Initial studies (Houghton, Houghton and Taylor, 1978) have shown that there is a prolonged increase in $^3$H-TdR uptake and utilization after FU administration in 3 FU insensitive tumour lines, but is decreased significantly in $ELC_2$ tumours. This may be due to pool-size perturbation, a genuine compensation for the inhibition of TMP production from dUMP, or possibly a cell-synchronizing effect of the treatment (Camplejohn, Schultze and Maurer, 1977).

It is anticipated that expansion of the current 6 colorectal xenograft lines will simulate a human tumour population, and that such a population will provide a more realistic screen for the selection of new agents, and in selecting drug combinations which will have significant activity clinically against a high proportion of colorectal tumours. Because tumour response to treatment is more readily defined in a xenograft system than in the patient, it may be possible to relate biochemical and biological parameters of the tumours to their chemosensitivity, eventually allowing for prediction of tumour sensitivity of individual patients, on the basis of correlations made in xenografts.

### REFERENCES

Ashwell, G. (1957) Colorimetric Analysis of Sugars. *Meth. Enzymol.*, **3**, 73.

Burton, K. (1956) A Study of the Conditions and Mechanisms of the Diphenylamine Reaction for Colorimetric Estimation of DNA. *Biochem. J.*, **62**, 315.

Camplejohn, R. S., Schultze, B. & Maurer, W. (1977). *In vivo* Cell Synchrony in the L1210 Mouse Leukaemia studied with 5-Fluorouracil or 5-Fluorouracil followed by Cold Thymidine Infusion. *Br. J. Cancer*, **35**, 546.

Carter, S. K. & Friedman, M. (1974) Integration of Chemotherapy into Combined Modality Treatment of Solid Tumors. II. Large Bowel Carcinoma. *Cancer Treatment Rev.*, **1**, 111.

DeVita, V. T. & Schein, P. S. (1973) The Use of Drugs in Combination for the Treatment of Cancer. *New Engl. J. Med.*, **288**, 998.

Houghton, P. J., Houghton, J. A. & Taylor, D. M. (1977) Effects of Cytotoxic Agents on TdR Incorporation and Growth in Human Tumour Xenografts. *Br. J. Cancer*, **36**, 206.

HOUGHTON, P. J., HOUGHTON, J. A. & TAYLOR, D. M. (1978) Factors Determining the Response to 5-Fluorouracil in Human Colonic Tumour Xenografts. *Br. J. Cancer*, **36**, 424.

HOUGHTON, J. A. & TAYLOR, D. M. (1978*a*) The Maintainance of Biological and Biochemical Characteristics of Human Colorectal Tumours During Serial Passage in Immune-deprived Mice. *Br. J. Cancer*, **37**, 199.

HOUGHTON, J. A. & TAYLOR, D. M. (1978*b*) Growth Characteristics of Human Colorectal Tumours During Serial Passage in Immune-deprived Mice. *Br. J. Cancer*, **37**, 213.

HOUGHTON, P. J. & TAYLOR, D. M. (1977) Fractional Incorporation of $^3$H-thymidine and DNA Specific Activity as Assays of Inhibition of Tumour Growth. *Br. J. Cancer*, **35**, 68.

KENT, R. J. & HEIDELBERGER, C. (1972) Fluorinated Pyrimidines XL. The Reduction of 5-Fluorouridine 5-diphosphate by Ribonucleotide Reductase. *Mol. Pharmac.*, **8**, 465.

KOPPER, L. & STEEL, G. G. (1975). The Therapeutic Response of Three Human Tumor Lines Maintained in Immune-deprived Mice. *Cancer Res.*, **35**, 2704.

KOVACH, J. S., MOERTEL, C. G., SCHUTT, A. J., REITEMEIER, R. G. & HAHN, R. G. (1973) Phase II Study of *cis*-Diamminodichloroplatinum in Advanced Carcinoma of the Large Bowel. *Cancer Chemother., Rep.*, **57**, 357.

LOWRY, O. H., ROSENBROUGH, N. J., FARR, A. L. & RANDALL, R. J. (1951). Protein Measurement with the Folin Phenol Reagent. *J. Biol. Chem.*, **193**, 265.

MADOC-JONES, H. & BRUCE, W. R. (1968) On the Mechanism of the Lethal Action of 5-Fluorouracil on Mouse L Cells. *Cancer Res.*, **28**, 1976.

MYERS, C. E., YOUNG, R. C. & CHABNER, B. A. (1975) Biochemical Determinants of 5-Fluorouracil Response *in vivo*: The Role of Deoxyuridylate Pool Expansion. *J. Clin. Invest.*, **56**, 1231.

NATHANSON, L., HALL, T. C., SCHILLING, A. C. & MILLER, S. (1969) Concurrent Combination Chemotherapy of Human Solid Tumors: Experience with a 3-drug Regimen and Review of the Literature. *Cancer Res.*, **29**, 419.

PICKARD, R. G., COBB, L. M. & STEEL, G. G. (1975) The Growth Kinetics of Xenografts of Human Colorectal Tumours in Immune-deprived Mice. *Br. J. Cancer*, **31**, 36.

POVLSEN, C. O. & JACOBSEN, G. K. (1975) Chemotherapy of a Human Malignant Melanoma Transplanted in the Nude Mouse. *Cancer Res.*, **35**, 2790.

REYES, P. (1969) The Synthesis of 5-Fluorouridine 5-phosphate by Pyrimidine phosphoribosyltransferase of Mammalian Origin I. Some Properties of the Enzyme from P1534J Mouse Leukemic Cells. *Biochemistry*, **8**, 2057.

ROFSTAD, E. K., BRUSTAD, T., JOHANNESSON, J. V. & MOSSIGE, J. (1977) Effects of Cobalt 60 Gamma Rays and DTIC (5-(3,3 dimethyl-1-triazene)-imidazole-4-carboxamine) on Human Malignant Melanoma Growth in Athymic Nude Mice. *Br. J. Radiology*, **50**, 314.

SNEIDER, T. W. & POTTER, V. R. (1969) Alternative *de novo* and "Salvage" Pathways to TTP Synthesis: Possible Implications for Cancer Chemotherapy. *Cancer Res.*, **29**, 2398.